# Spitz nevus and infliximab: association or coincidence?^☆^^☆☆^

**DOI:** 10.1016/j.abd.2020.01.008

**Published:** 2020-07-12

**Authors:** Catarina Soares Queirós, André Laureano-Oliveira, Dolores Lopéz-Presa, Paul Filipe

**Affiliations:** a Dermatology Department, Hospital Santa Maria, Centro Hospitalar e Universitário de Lisboa Norte, Lisbon, Portugal; b Faculdade de Medicina de Lisboa, Lisbon, Portugal; c Pathology Department, Hospital de Santa Maria, Centro Hospitalar e Universitário de Lisboa Norte, Lisbon, Portugal

**Keywords:** Biological agents, Confocal microscopy, Dermoscopy, Nevus, epithelioid and spindle cell, Skin neoplasms

## Abstract

Biological therapies, including anti-TNF agents, are important in the treatment of various chronic inflammatory diseases, including psoriasis, rheumatoid arthritis or inflammatory bowel disease. The increased use of these drugs translates into an increasing awareness of its adverse effects, which include malignancy. In this paper, we describe the case of a 28-year-old woman who developed a spitzoid melanocytic tumor after starting infliximab therapy for ulcerative colitis. The evidence for causality between anti-TNF and melanocytic proliferations is still sparse; nonetheless, treatment-associated immunosuppression seems to play a key role in this phenomenon. Therefore, a regular follow-up with a rigorous skin examination is essential in these patients. Noninvasive techniques such as dermoscopy or reflectance confocal microscopy are particularly useful diagnostic tools in these circumstances.

## Introduction

Tumor necrosis factor alpha (TNF-α) is a cytokine of the innate immune system with important functions in the regulation of systemic inflammation and the detection of malignancies and infections.^1^, ^2^ Therefore, its inhibition can improve several dermatological, rheumatological, and gastrointestinal inflammatory disorders, making these agents important in the treatment of many chronic inflammatory diseases.^2^

With the increasing usage of these drugs, adverse effects have been increasingly recognized, such as the increased risk of infection and malignancies. Long-term data for anti-TNF agents regarding the risk for the development of malignancies are lacking, but recently several case reports that associate anti-TNF therapies with melanoma have been published.^1^, ^2^

## Case report

**Figure 1:**
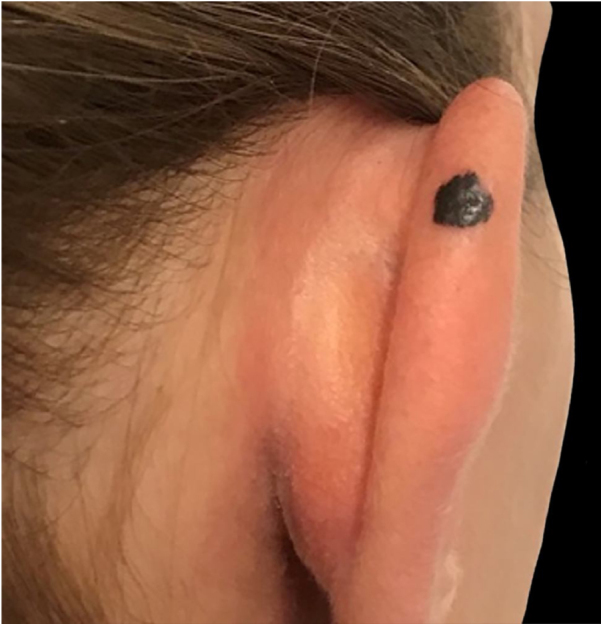
Spitz nevus: clinical photography. Brown macule with a diameter of 6 mm on the right helix.

**Figure 2:**
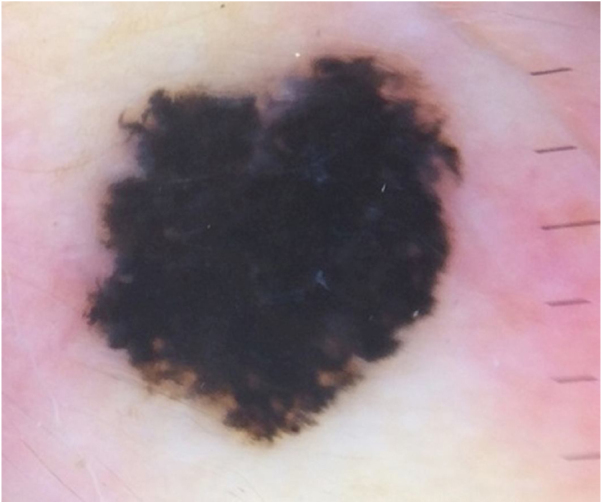
Spitz nevus: digital dermoscopy. Melanocytic lesion with a star-burst type global pattern.

**Figure 3:**
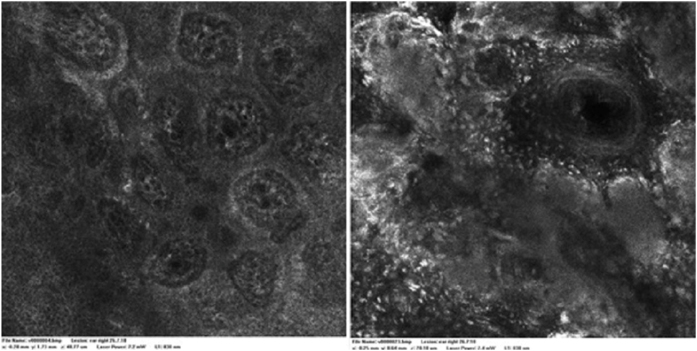
Spitz nevus: reflectance confocal microscopy. Well-delimited regular nests at the dermo-epidermal junction and pagetoid infiltration. (VivaScope1500, A and B basic images 0.5 × 0.5 mm).

**Figure 4:**
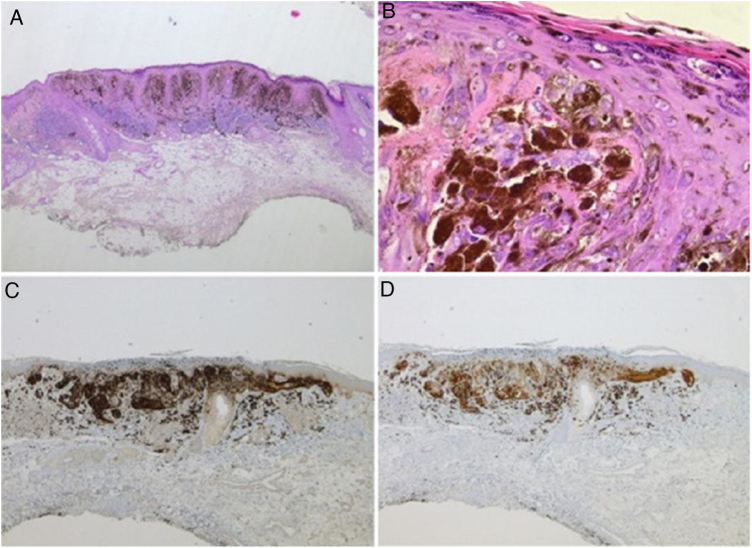
Spitz nevus: histopathological examination. A, Compound symmetric melanocytic lesion, consisting of large epithelioid and fusiform melanocytes, with many melanophages; acanthosis and irregular hyperplasia of the epidermis (Hematoxylin & eosin, ×25). B, Kamino bodies (Hematoxylin & eosin, ×100). C, Positive immunohistochemistry for HMB45 (×40). D, Positive immunohistochemistry for p16 (×40).

A 28-year-old woman, Fitzpatrick skin phototype 2, with a three-year history of ulcerative colitis, was referred to the Department of Dermatology due to a pigmented lesion on her right ear. She had been under infliximab therapy for the last 30 months. The lesion had appeared six months earlier and had progressively grown. On examination, a dark brown macule on the helix was evident, with regular borders and a diameter of 6 mm (Fig. 1). There were no palpable lymphadenopathies. Dermoscopy revealed a melanocytic lesion, with a global starburst pattern, suggestive of spitzoid melanocytic tumor (Fig. 2). Examination with reflectance confocal microscopy (RCM) showed a well-demarcated lesion, with junctional nests and pagetoid infiltration (Fig. 3). The lesion was surgically excised and histological examination confirmed a compound Spitz nevus (Fig. 4). A decision to maintain therapy with infliximab was made. Sun-protective measures were emphasized, and the patient remains under close clinical follow-up.

## Discussion

TNF-α is a cytokine of the innate immune system with a critical function in the surveillance of malignancies and infections.^3^ TNF-α antagonists are a group of biologic agents that include infliximab, etanercept, and adalimumab, and are currently indicated in the treatment of several inflammatory conditions including psoriasis and inflammatory bowel disease.^3^

The most common adverse effects of this group of biologic agents are in general mild. However, serious complications such as infections or malignancies have been reported.^3^, ^4^ In the last two decades, several case reports have pointed to an overall increased risk of malignancy, namely lymphoma and non-melanoma skin cancer (NMSC) in patients treated with these agents.^5^ NMSC in particular seems to be associated with chronic immunosuppression, accounting for up to 95% of post-transplant cutaneous malignancies.^5^ More recently, the association between these anti-TNF antagonists and the appearance of melanoma has also been described.^2^, ^5^, ^6^

The potential impact of TNF antagonists on the development of skin cancer occurs in two ways. First, these drugs may affect tumor initiation, altering the incidence of malignancies; secondly, they may reduce the immunologic control of mutated cells or pre-existing subclinical tumors, allowing for rapid tumor growth.^6^

The evidence for causality between anti-TNF and the development of melanocytic proliferations is still sparse, and the possible link between biological therapy and the induction of melanocytic proliferation needs further illumination.^4^ Melanocytic proliferation may be benign, as in eruptive melanocytic nevi, or malignant, as in melanoma. Eruptive nevi are a rare phenomenon consisting of an abrupt appearance of multiple nevi or large atypical lesions simulating melanoma. Its occurrence has been associated not only with dermatological diseases but also with immunosuppression.^7^

It is believed that immunosuppression may induce melanocyte-stimulating hormone or melanoma growth-stimulatory activity, two endogenous growth factors for melanocytes.^4^ Therefore, growth and development of melanocytes is stimulated under these circumstances. Genetic factors may also be involved. The role of immunosurveillance in tumorigenesis is therefore essential, and a diminished immunosurveillance can lead to the appearance of malignant lesions or to malignant transformation of benign lesions.^4^

The appearance of Spitz nevus in anti-TNF treated patients has been reported. Sousa reported a 29-year-old woman who developed a dysplastic spitzoid compound naevus on her right thigh after 41 months of psoriasis treatment with adalimumab.^8^ Although they are benign skin tumors, Spitz nevi may resemble malignant melanomas both clinically and microscopically. Therefore, these lesions are commonly excised for precaution. In the present case, the patient developed a compound Spitz nevus in a non-exposed area after 24 months of infliximab treatment. Although fair skin type may have been a contributing factor to the development of this lesion, the immunosuppressive effects of infliximab may also have played a role.

Spitz nevi usually display a dynamic behavior, with considerable variation in their morphology, dermoscopy, and RCM features over time.^9^ Observation with RCM usually shows a symmetric lesion, frequently with pagetoid infiltration, especially as spindle cells throughout the lesion. Oval to round polygonal nests with well-defined borders, composed by clustered cells, frequently large in size and highly reflective, are also characteristic features. Regarding the main RCM pattern, most Spitz nevi show a clod pattern or an association of clod to meshwork or ringed pattern.^9^

While dermoscopy is an established tool in assisting noninvasive diagnosis in dermatology, RCM is still acquiring its role in clinical practice. In this setting, it may be particularly useful for the assessment of melanocytic lesions identified as “equivocal” by visual inspection or dermoscopy. Here, the available evidence suggests that RCM may be both more sensitive and specific in comparison to dermoscopy, which underscores the potential of this imaging method in reducing the number of inappropriate excisions.^10^ However, it should be emphasized that all spitzoid lesions in adults should be clarified from a histological point of view.

In sum, although additional studies are needed to better characterize the impact of biologic therapy in melanocyte proliferation, there is growing evidence suggesting a possible relation between biological therapy and melanocytic proliferation.^4^ Controlled studies with larger sample sizes and longer follow-up periods are needed in order to better evaluate this association. Until then, the available literature suggests that patients undergoing treatment with biologics should be encouraged to monitor their pre-existing nevi as well as the appearance of new ones.^6^, ^8^ Regular observation by a dermatologist should also be advised. In following these patients, non-invasive imaging techniques have acquired a special role as a means of detecting subtle changes and monitoring evolution of the lesions.

## Financial support

None declared.

## Authors’ contributions

Catarina Sousa Duque Soares Queirós: Approval of the final version of the manuscript; conception and planning of the study; drafting and editing of the manuscript; collection, analysis, and interpretation of data; participation in study design; intellectual participation in the propaedeutic and/or therapeutic conduct of the studied cases; critical review of the literature; critical review of the manuscript.

André Laureano Oliveira: Approval of the final version of the manuscript; critical review of the literature; critical review of the manuscript.

Dolores Lopéz Presa: Approval of the final version of the manuscript; critical review of the literature; critical review of the manuscript.

Paulo Filipe: Approval of the final version of the manuscript; critical review of the literature; critical review of the manuscript.

## Conflicts of interest

None declared.
